# Preface to the third issue of Journal of Cardiovascular Disease Research

**DOI:** 10.4103/0975-3583.70897

**Published:** 2010

**Authors:** Yujie Zhu

**Affiliations:** *Associate Editor, Journal of Cardiovascular Disease Research, Birmingham, AL, USA*

We are delighted to present to you the third issue of the *Journal of Cardiovascular Disease Research (JCDR)*. We would like to take this opportunity to extend our appreciation to our valued readers and authors for their continuing interest in *JCDR*, and to every member of the editorial board and review board for their dedication to this scientific endeavor, especially Drs. Cuilan Li, Jing Dai, Jiusheng Deng, Zhenquan Jia, Ningpu Yu, Nan Zhang, Yueting Shang, Elizabeth Gilbert, Qiong Gan, and Peng Liang who also serve as executive editors to expand the influence and quality of the journal.

With the publication of its third issue, the *JCDR* is poised for continued growth, progress, and a bright and vibrant future. It is encouraging that we are receiving both a higher quality and greater volume of manuscript submissions. The articles published in the third issue cover a broad range of topics in cardiovascular disease research.

In the Invited Review, Dr. Gao *et al*. summarized our current understanding of high-density lipoprotein (HDL) functions and HDL-targeted pharmacotherapy. This paper is more focused on the mechanistic issues, providing extensive details on pathways underlying HDL beneficial functions and drug mechanisms. The author reviewed the current status of clinically used HDL-based therapies and other therapies that are still in development. The author highlighted the new direction for HDL-based therapies, including development of functionally improved HDL as an alternative to simply increasing HDL-C level.

Dr. Li *et al*. evaluated the effects of high dose glucose–insulin–potassium solution on hemodynamics and cardiac remodeling in patients with acute myocardial infarction (AMI) treated with primary percutaneous coronary intervention (PCI). The authors found that high dose glucose–insulin–potassium solution had no adverse effects on hemodynamics in AMI patients treated with primary PCI. They observed that it could improve cardiac function. Their findings provide important information for healthcare professionals.

Dr. Sliem *et al*. confirmed the increased aortic stiffness in patients with rheumatoid arthritis. Meanwhile, they also found that multiple clinical factors, including age, duration of rheumatoid arthritis, smoking index, waist circumference, and triglyceride and C-reactive protein levels were significantly higher in patients with aortic stiffness.

The study by Dr. Nasr *et al*. examined the relationship between silent ischemia and insulin resistance in prediabetic patients. The authors hypothesized that glucose intolerance and insulin resistance in prediabetic adults could be risk factors for coronary heart disease. They concluded that prediabetics have myocardial perfusion defects that pose a cardiovascular risk, which are associated with insulin resistance and visceral obesity but are independent of fasting glucose levels.

Das *et al*. evaluated the association of metabolic syndrome with obesity measures, metabolic profiles, and intake of dietary fatty acids. The data suggest that saturated fat intake may be a major risk factor for the onset of metabolic syndrome.

The paper by Taliyan and colleagues reported that activation of α_1_ adrenergic receptors and K_ATP_ channels may be responsible for the cardioprotective effect of remote aortic preconditioning in isolated rat heart.

Dhar *et al*. studied the genetic and environmental interactions related to coronary artery disease. Their study suggests that methylenetetrahydrofolate reductase gene polymorphisms have significant association with coronary artery disease.

There are other interesting papers in the third issue. These articles discussed other hot topics in the area of cardiovascular disease.

Looking to the future, we will continue to deliver the best of recent ideas and developments in cardiovascular disease research and publish high-impact findings of the broadest significance to the field. We are working on a process to optimize the online system for peer review. We welcome your feedback on how our journal is doing and how we can make it even more useful to you. As always, we say thank you for your support and we look forward to a successful future for JCDR.

